# The Story of the Insane from Year to Year

**Published:** 1897-12-04

**Authors:** 


### THE STORY OF THE INSANE FROM YEAR TO YEAR.

Tenth Series.

### MULLINGAR DISTRICT ASYLUM.

The limits of accommodation are given at 621, but there were 712 in residence at the close of the year 1896. The first admissions were 149, the readmissions 46, the recoveries 72, and the deaths 36. The recoveries stand at 38·9 per cent. of the admissions, and the deaths at 5·2 of the average number resident. There were only 10 deaths from cerebral diseases, and general paralysis does not figure in the returns at all; but when we come to thoracic diseases, we find that 16 of the deaths were caused by consumption. Eighteen of the year's admissions were due to moral causes, 13 to intemperance in drink, and 59 to hereditary influences. A block for 130 patients is about to be built, but in view of the present overcrowding and the probable annual increment of patients, it is to be feared that the contemplated additions are not nearly extensive enough. Dr. Finegan does not think that the increase in the asylum population is owing to an actual increase of insanity in the district, but rather to the diminished death-rate and to the drafting of chronic lunatics from the workhouses. In spite of the lamentable overcrowding, the asylum would seem to be managed by an enlightened committee, for we learn that a new water supply has been provided, the drainage system remodelled, and the electric light introduced.

